# Rationale and study protocol of ACQUIRE, a prospective, observational study measuring quality of life, treatment preference and treatment satisfaction of autosomal dominant polycystic kidney disease (ADPKD) patients in Europe

**DOI:** 10.1186/s12882-020-01927-1

**Published:** 2020-07-24

**Authors:** Dominique Joly, Jennifer Quinn, Stella Mokiou, Karl O’Reilly, Joaquín Sánchez-Covisa, Jing Wang-Silvanto, Helen Doll

**Affiliations:** 1 Nephrology Department, Necker Hospital, 149 Rue de Sèvres, 75015 Paris, France; 2 BioMarin Pharmaceutical Inc., London, UK; 3 Otsuka Pharmaceutical Europe Ltd., Wexham, UK; 4 Clinical Outcomes Solutions, Folkestone, UK

**Keywords:** ADPKD, Discrete choice experiment (DCE), Quality of life, Tolvaptan, Aquaresis, Observational

## Abstract

**Background:**

Autosomal dominant polycystic kidney disease (ADPKD) is considered the most common inherited renal disease. Patient-Reported Outcomes (PROs) and patient experience in ADPKD are difficult to quantify and have not been well studied, particularly in the early stages of the disease. There is evidence to suggest that early-stage ADPKD patients have a lower Health-Related Quality of Life (HRQoL) than the general population due to the signs and symptoms of early-stage ADPKD. However, no research has been carried out on the HRQoL of early-stage ADPKD patients using validated ADPKD-specific PRO measures. Additionally, a new disease progression delaying treatment option has recently emerged for ADPKD. Patient preference for this treatment and unmet treatment needs have not yet been investigated.

**Methods:**

The ACQUIRE study is a prospective, observational study investigating the influence of early-stage ADPKD-related symptoms and treatments on PROs. It aims to collect real-world data on patient demographics, treatment patterns, clinical outcomes, and PROs such as HRQoL, treatment satisfaction and treatment preference in early-stage ADPKD. Adult ADPKD patients in stages 1–3 of chronic kidney disease (CKD) with evidence of rapidly progressing disease are being recruited from seven European countries. At baseline and every 3 months, for a follow-up period of 18 months, general and disease-specific questionnaires are completed remotely to capture patients’ own assessment of their overall and ADPKD-related HRQoL. A Discrete Choice Experiment (DCE) is also used to investigate the value patients place on different attributes of hypothetical treatment options (e.g. treatment outcomes, side effects) and the role each attribute plays in determining overall patient treatment preference.

**Discussion:**

The results of this study will highlight the real-world effects of ADPKD-related challenges on PROs including HRQoL, treatment experience and satisfaction; and help physicians gain greater insight into likely disease outcomes based on early-stage patient symptoms and patients’ experience with treatment. Data captured by the DCE may inform ADPKD treatment decision-making from a patient perspective. The DCE will also provide insights into which patients are more likely to perceive benefit from treatments based on the value and trade-offs they place on specific treatment attributes.

**Trial registration:**

NCT02848521.

**Protocol Number/Version**: 156–303-00096/Final

**World Health Organization Trial Registration Data Set**

**Table:** 

Data category	Information
Primary registry and trial identifying number	ClinicalTrials.gov
Date of registration in primary registry	July 21, 2016
Secondary identifying numbers	156–303-00096
Source(s) of monetary or material support	Otsuka Pharmaceutical Europe Ltd
Primary sponsor	Otsuka Pharmaceutical Europe Ltd
Secondary sponsor(s)	N/A
Contact for public queries	Study Director, Medical Department Otsuka EuropeTel: + 44 (0) 2037475000
Contact for scientific queries	Study Director, Medical Department Otsuka EuropeTel: + 44 (0) 2037475000
Public title	A Study Measuring Quality of Life, Treatment Preference and Satisfaction of ADPKD Patients in Europe
Scientific title	A Prospective, Non-interventional Study Measuring Quality of Life, Treatment Preference and Treatment Satisfaction of Autosomal Dominant Polycystic Kidney Disease Patients in Europe
Countries of recruitment	Austria, Belgium, France, Germany, Spain, Switzerland, United Kingdom
Health condition(s) or problem(s) studied	Autosomal Dominant Polycystic Kidney Disease
Intervention(s)	N/A – this is a non-interventional study
Key inclusion and exclusion criteria	***Inclusion Criteria:*** Male and female aged ≥18 years. Patient has a diagnosis of ADPKD between CKD Stages 1–3, and is deemed by their treating physician to likely have rapidly progressing disease. Patient has a life expectancy greater than 18 months at time of enrolment. Patient is able and willing to give informed consent, if required according to local regulations. Patient is fluent in local language. ***Exclusion Criteria:*** Patient is currently participating in or has in the last 12 months participated in an interventional clinical trial. Presence of any condition/circumstance which in the opinion of the investigator could significantly limit the complete follow up of the patient. Inability of the patient to complete PROs remotely.
Study type	Observational
Date of first enrolment	October 2016
Target sample size	486
Recruitment status	Active, not recruiting
Primary outcome(s)	Mean rate of change (%) in Physical Health Composite Scale (PCS) scores of the SF-12 from baseline to end of study, in the overall sample and per chronic kidney disease (CKD) stage.
Key secondary outcomes	Mean rate of change (%) in Mental Health Composite Scale (MCS) scores of the SF-12 from baseline to end of study, in the overall sample and per CKD stage. Mean ADPKD-IS scores changes from baseline to end of study, in the overall sample and per CKD stage (physical, emotional and fatigue domain scores will be reported and analysed). Mean TSQM-9 score changes from baseline to end of study, in the overall sample and per CKD stage (effectiveness, convenience and global satisfaction domain scores will be reported and analysed). Mean ADPKD-UIS score changes from baseline to end of study, in the overall sample and per CKD stage (frequency, urgency and nocturia domain scores will be reported and analysed). Mean ADPKD-PDS score change from baseline to end of study, in the overall sample and per CKD stage (dull kidney pain, sharp kidney pain and fullness/discomfort domain scores will be reported and analysed) Overall odds ratio of discrete-choice experiment (DCE) for patient preference to the addition of a disease modifying treatment versus no change to local Standard of Care (SoC) from baseline to end of study, in the overall sample and per CKD stage

## Background

Autosomal dominant polycystic kidney disease (ADPKD) is the most common inherited renal disease, affecting 3.29/10,000 people in Europe [1, 2]. ADPKD is caused by genetic mutations in one of two genes: polycystin 1 (PKD1) or polycystin 2 (PKD2), with approximately 10% of cases caused by an apparent de novo mutation [3]. The disease is characterized by the progressive increase in the number and size of bilateral renal cysts, causing hypertension, kidney pain and eventually kidney failure [4–6]. Symptoms of ADPKD can range from mild to severe, and worsen as disease progresses. Early symptoms can include: abdominal pain, high blood pressure, hematuria, increased vulnerability to urinary tract infections, nephrolithiasis, and urine concentrating defects [4, 7]. ADPKD is a leading cause of end stage renal disease (ESRD); by the age of 60 approximately 50% of ADPKD patients will progress to ESRD and will require either dialysis or a kidney transplant. ADPKD accounts for 5–10% of all ESRD patients [8].

Until recently, all treatments targeted the signs and symptoms of ADPKD, such as high blood pressure and pain, as a patient progressed to ESRD [9]. Tolvaptan, a vasopressin V2 receptor antagonist, is the first licensed ADPKD treatment shown to slow the progression of cyst development and renal insufficiency [10]. Tolvaptan promotes aquaresis and fluid loss, reducing the amount of fluid stored in the kidneys. Additionally, it reduces cyclic adenosine monophosphate (cAMP) levels, reducing the rate of kidney cell cyst proliferation [11]. Treatment with tolvaptan during early-stage chronic kidney disease (CKD) has been shown to slow total kidney volume growth and glomerular filtration rate (GFR) decline [10]. Based on clinical trial data, long-term treatment models indicate tolvaptan may delay ESRD by up to 6.5 years [12]. However, tolvaptan treatment can lead to hepatotoxicity and higher doses are not well tolerated due to a range of side effects, including aquaresis and polydipsia. In order to limit the impact of side effects, a large number of patients take a maximum tolerated dose, rather than the highest dose available, however the effect of tolvaptan on patient quality of life (QoL) has not yet been investigated [13, 14].

There is limited evidence to suggest that the QoL of patients with ADPKD is significantly lower than that of the general population. For example, a study in Japan found ADPKD patients scored significantly lower than the general population on physical, mental and social component summary scores of the health-related QoL (HRQoL) Short Form-36 (SF-36) questionnaire [2]. Previous research on HRQoL has mainly focused on the later stages of ADPKD (e.g. stages 4–5) and available data are derived from monocentric, cross-sectional studies conducted prior to the availability of tolvaptan [2]. A small number of studies have investigated QoL in early-stage ADPKD (CKD stages 1–3) finding that, while QoL is higher in early stages of the diseases than during the later stages, early-stage ADPKD has significant physical and emotional impact on patients [15, 16]. However, these studies used generic measures of HRQoL, rather than questionnaires specifically designed to assess the disease-specific effects ADPKD has on patients’ HRQoL. Thus, these findings may not present an accurate picture of the burden of the disease.

The ACQUIRE study is a multi-center, prospective, observational study investigating the real-world impact of ADPKD on patients at the early stages of the disease who have shown indications of rapidly progressing kidney dysfunction. This study aims to use ADPKD disease-specific measures to assess patients’ HRQoL in the early disease stages and as disease progresses. These findings may then be used to develop prognostic markers and treatment response prediction models. This study also aims to measure patient satisfaction and treatment preference using a Discrete Choice Experiment (DCE).

## Methods

### Study design

**Fig. 1:**
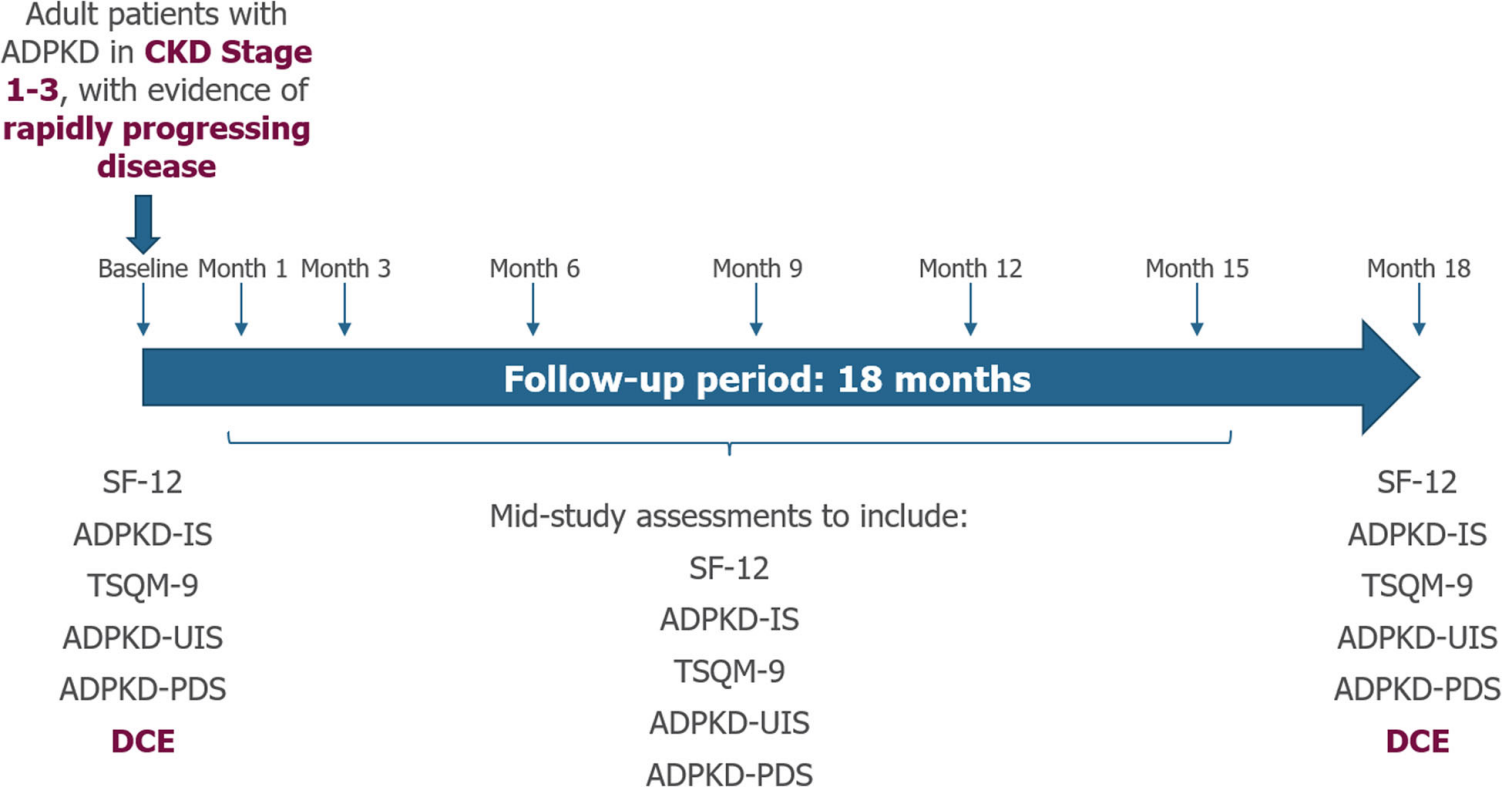
Study Design. ADPKD: autosomal dominant polycystic kidney disease; ADPKD-IS: ADPKD Impact Scale; ADKPD-PDS: ADPKD-Pain and Discomfort Scale; ADPKD-UIS: ADPKD-Urinary Impact Scale; CKD: chronic kidney disease; DCE: discrete choice experiment; SF-12: 12-item Short Form Health Survey; TSQM-9: Abbreviated Treatment Satisfaction Questionnaire for Medication

The ACQUIRE study is a prospective, observational study aiming to measure HRQoL, treatment preference and satisfaction and other patient reported outcomes (PROs) of ADPKD patients in Europe (Fig. 1). Data are prospectively collected at clinics, from medical notes and via PRO instruments for each patient at Baseline, Month 1, Month 3, and subsequently at 3-month intervals up to and including the final assessment (18 months maximum follow-up time). All data collection is expected to finish by the end of October 2019. Patients have been recruited from seven European countries: Austria, Belgium, France, Germany, Spain, Switzerland and the United Kingdom (UK).

### Patient inclusion and exclusion criteria

Adult patients (≥18 years) with ADPKD in CKD stages 1–3, with evidence of rapidly progressing disease, and a life expectancy of longer than 18 months at the time of enrolment, were recruited to this study. These inclusion criteria are in line with the criteria for tolvaptan use in Europe [17]. The classification of rapid progression was left to the discretion of the doctor. European experts have proposed four ways to identify patients with evidence or risk of rapid ADPKD progression: [i] GFR slope, [ii] kidney growth rate, [iii] predictive model based on renal volume or renal length, or [iv] predictive model based on genetics [18]. All respondents were fluent in the local languages and were able and willing to give informed consent.

Individuals were excluded from the study if they were currently participating in, or had in the last 12 months participated in, an interventional clinical trial. Additional exclusion criteria included the presence of any condition/circumstance that, in the opinion of the investigator, could limit a complete 18-month study follow-up and prevent patients from completing the PRO questionnaires remotely. Based on a previous large study on a similar population, it was expected that the number of patients in each CKD stage would be approximately equal, so no restriction based on the number of patients at each early CKD stage was imposed during the recruitment stages (OVERTURE Study, NCT01430494) [19].

### Key study outcomes

The primary objective of this study is to describe the HRQoL of adult ADPKD patients in CKD stages 1–3 with rapidly progressing disease, overall and per CKD stage.

Secondary objectives of the study are to describe patient demographics, treatment satisfaction, the burden of aquaresis, and real-world ADKPD treatment patterns of adult ADPKD patients in CKD stages 1–3 with rapidly progressing disease, overall and per CKD stage.

This study also aims to investigate a range of exploratory outcomes. Specifically, change in patient reported pain scores from baseline (mean changes in dull kidney pain, sharp kidney pain and fullness/discomfort scores from the ADPKD Pain and Discomfort Scale [ADPKD-PDS]). The DCE will explore patient preferences for adding a disease modifying treatment versus no change to local standard of care, with the analysis exploring the relationships between treatment attributes and overall treatment preference. In addition, any relationship between DCE attribute preferences and persistence to ADPKD treatments will be investigated. Finally, exploration of possible relationships between variables of interest that could predict PRO instrument scores and other outcomes like persistence to ADPKD treatments may be carried out using Structural Equational Modelling (SEM). If SEM analyses are completed, the variables will be selected based on patient characteristics and potential differences in these between patient subgroups.

The primary endpoint assessed to measure HRQoL is the physical component score (PCS) of the 12-item Short Form Health Survey (SF-12) HRQoL questionnaire. The secondary endpoints include the mental component score (MCS) of the SF-12, the TSQM-9 and the disease-specific ADPKD-IS and ADPK-UIS. For both primary and secondary endpoints the outcome values and change from baseline values at each assessment, as well as the percentage change (%) from baseline to end of study measurements are recorded. These data will be presented for the overall sample, and for each CKD stage.

### Study procedures and measurements

Data are collected prospectively during clinic visits, from medical notes and from electronic questionnaires via a web-based data capture system that patients complete remotely. As this is an observational (real-world) study, patients are not required to attend additional clinic appointments; all data are collected during routine clinic visits.

At study baseline, patient demographics, medical history and current medication (both ADPKD-related and concomitant) are recorded. Additionally, medical outcomes (including patient reported adverse events), medication, and some PROs are collected at baseline and mid-study assessments at Month 1, Month 3 and then at 3-month intervals up to and including 18 months (Fig. 1). These PROs include HRQoL (measured by the SF-12 and ADPKD Impact Scale [ADPKD-IS]), treatment satisfaction (measured by the abbreviated Treatment Satisfaction Questionnaire for Medication [TSQM-9]), burden of aquaresis on patients (the ADPKD-Urinary Impact Scale [ADPKD-UIS]), and pain (the ADPKD-Pain and Discomfort Scale [ADPKD-PDS]). Patient treatment preferences (measured by DCE) are collected at baseline and during the final study visit. An overview of each questionnaire is provided below.

#### Patient questionnaires

General and disease-specific questionnaires are used to capture the patients’ assessment of their HRQoL, both broadly and with reference to specific symptoms and challenges faced when living with ADPKD. Questionnaires are completed remotely to allow flexibility and to ensure the information collected is reflective of real-world patients’ experience. Reminders to complete the questionnaire are sent via email at each timepoint to maximize questionnaire completion.

The SF-12 is a validated short questionnaire capturing the patient’s assessment of their functional health and well-being [20]. It has been developed as a shorter alternative to the SF-36 and covers 8 domains of HRQoL: physical functioning, role-physical, bodily pain, general health, vitality, social functioning, role-emotional, and mental health [21]. This study uses the SF-12 version 2 (4-week recall). The questionnaire is weighted and summed to provide easily interpretable scales of physical and mental health (PCS and MCS, respectively). Each scale is computed using scores from the 12 questions and scores range from 0 to 100, where 0 indicates the lowest level of HRQoL.

The TSQM-9 is a validated measure of patient satisfaction with medication and includes 3 domains: effectiveness, convenience, and global satisfaction [22]. It is based on the Treatment Satisfaction Questionnaire for Medication (TSQM) Version 1.4 and includes 9 of the 14 questions from this longer questionnaire (the TSQM-9 does not include questions about the side effects of medication) [23]. Similar to the SF-12 scales, the TSQM-9 domains are scored from 0 to 100, with higher scores representing greater satisfaction.

The ADPKD-IS measures the impact of ADPKD on a patient’s HRQoL using 3 domains: physical, emotional, and fatigue.^13, 14^ Each domain is measured by summing the score for each item in the domain and dividing the total number by the number of items in that domain. Each domain score is measured on a scale of 1–5, from 1, not difficult/bothered at all, to 5, extremely difficult/bothered, with a recall period of 14 days.

The ADPKD-UIS captures the burden of urinary concerns in ADPKD using 11 items that assess 3 domains: daytime urinary frequency, daytime urinary urgency, and nocturia [24]. ADPKD-UIS measures each domain by summing the score for each item in the domain and dividing it by the number of items in that domain. Each domain score is measured on a scale of 1–5 where 1 is not difficult/bothered and 5 is very difficult/bothered. The questionnaire has a recall period of 7 days.

Finally, the ADPKD-PDS measures the impact of three types of ADPKD-related pain: dull kidney pain, sharp kidney pain, and fullness/discomfort [25]. Each domain is measured on a 5-point scale, where 1 is no pain/discomfort and 5 is extreme pain/discomfort, with a recall period of 7 days.

#### DCE study

During the first and last study visits, a DCE questionnaire is administered to determine how highly patients value specific attributes of hypothetical alternative treatment options. A DCE is a health economic methodology used to explore the relative importance of each treatment attribute, helping to inform future treatment decisions [26]. DCE studies are used to quantify patient preferences for specific treatment attributes, and to investigate the role each treatment attribute plays in determining patient’s overall preference for a treatment [27]. Patients are offered a choice of hypothetical alternative treatments that are defined by different and varying attributes. Patients are asked to compare these hypothetical treatments and choose which they prefer. The analysis of patient responses across a number of different discrete treatment choices allows individual attributes to be ranked in order of patient preference.

**Fig. 2:**
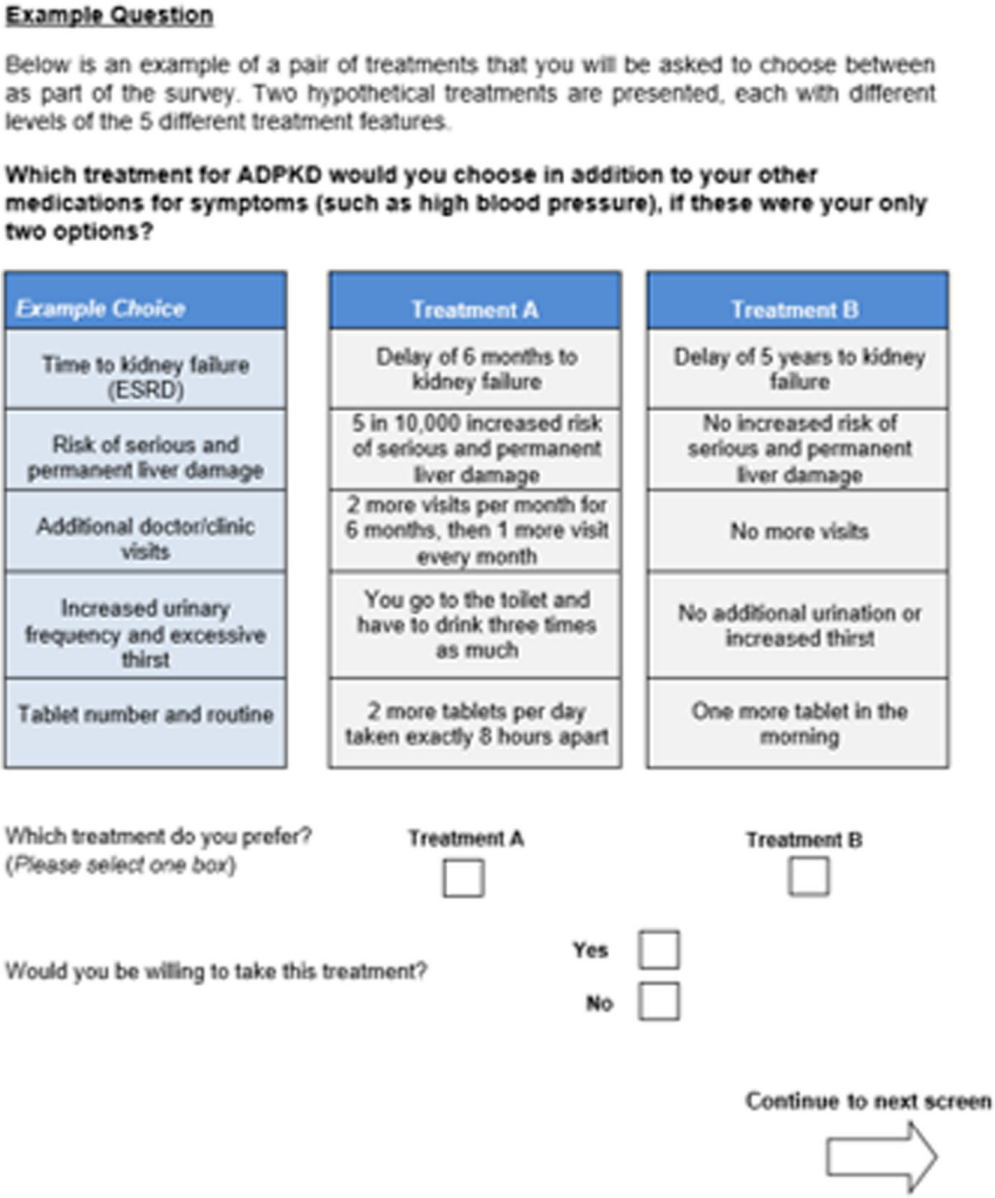
Example DCE Question. ADPKD: autosomal dominant polycystic kidney disease; ESRD: end stage renal disease

The DCE used in this study was developed to measure patient-perceived relative importance of different treatment attributes (aquaresis, time to kidney failure, risk of serious and permanent liver damage, additional doctor/clinic visits, tablet number and routine). Patients are asked to assigned each attribute to one of 3 levels ranging from equal to the effects of local standard of care (e.g. ‘no additional urination or increased thirst’) to very different from the effects of local standard of care (e.g. ‘you have to go to the toilet and have to drink three times as much’) (Fig. 2). The orthogonal design (where the occurrence and level of each attribute is unrelated to any other attribute) of the DCE allows the assessment of the role and importance of each attribute in determining treatment preference from patient responses. The outcome is expressed as an odds ratio. Cognitive debriefing interviews and a steering committee of experts were used to validate the DCE study design, and a pilot test of the study was performed in a small number of patients (*n* = 5), which demonstrated that the study was internally and externally consistent [28].

### Statistical analysis

#### Sample size calculation

This is a descriptive, observational study without requirement to power the sample for statistical inference. However, the statistical power for measurement accuracy (i.e. changes of the physical health scale measured by SF-12 from baseline) has been carried out for sample size estimation. This was based on observed variation of the physical health scale in the SF-12 questionnaire in a previous study on a similar population (OVERTURE Study, NCT01430494) [19]. The primary endpoint of the ACQUIRE study is the percentage change between baseline and throughout the observational points in the PCS of the SF-12 questionnaire. Assuming a loss of follow-up of 40% (similar to the 37% rate in the first 18 months of the OVERTURE study), a sample size of 162 patients per CKD stage (486 patients total) allows the detection of an average change of 1.4 points in PCS from baseline to the end of the study with 95% confidence interval (assuming a similar standard deviation [SD] to the OVERTURE study [SD = 7.0]). A 2.5 point change is considered clinically relevant and important in this disease area, so this sample size is sensitive enough to detect clinically relevant changes (1.4 point and above) in patients’ HRQoL [29]. Therefore, the target sample size has been defined as 480 patients. It is expected that approximately 20–25 centers, with 15–30 patients per center will provide the representative sample required in the study.

#### Statistical analysis of data

Data will be summarized using summary statistics. For continuous data the number of observations, numbers of missing data points, the mean, standard deviation, minimum, first quartile, median, third quartile and maximum will be reported. Counts and percentages will be presented for categorical data. Real-world treatment patterns for ADPKD patients, both overall and CKD stage, will be reported in this way.

The primary efficacy analysis is the change in PCS on the SF-12 questionnaire. For each visit the PCS will be summarized. The absolute values, change from baseline values and mean percentage change from baseline will be reported overall and for each CKD stage. The percentage change will be calculated as follows:

$$ Percentage\ change=\frac{100\times \left( score\  at\  end\  of\ study- score\  at\  baseline\right)}{score\  at\  baseline} $$

The secondary efficacy analysis is the change in the MCS on the SF-12 questionnaire. For each visit, overall and for each CKD stage the MCS will be summarized by the absolute values, change from baseline values and mean percentage change from baseline. The percentage change will be calculated as described above.

The absolute values, change from baseline values and mean percentage change from baseline will be reported for each CKD stage and overall for each questionnaire, at each timepoint. As an exploratory outcome, SEM analysis may be used to identify relationships between variables that could predict PRO scores and other outcomes, such as persistence, the act of continuing treatment for the prescribed duration [30]. Any hypotheses will be developed prior to the analysis of the final data set, but will be based on patient characteristics as baseline. Until baseline results are available, factors that may differ between patient subgroups and any resulting SEM hypotheses cannot be fully defined.

The DCE will be analyzed using mixed effect and multinomial logit models, exploring the impact of each treatment attribute on patient choice [31]. Specifically, two relationships will be studied: [i] the relationship between attributes of treatment and overall treatment preference, as captured in the DCE, and [ii] relationship between stated DCE attribute preferences and persistence to ADPKD treatments. Using results from a multinomial logit model, marginal rates of substitution will be calculated to measure how patients trade-off between certain attributes in their decision making; preference heterogeneity will be investigated by incorporating interactions between individual characteristics (such as gender, age, and disease severity) and relevant levels.

No imputation methods will be used to replace missing data; incomplete or missing data will be left as recorded. However, if a date is required for analysis or to define subgroups, the most conservative approach will be used. For example, if an adverse event has occurred, it will be assumed that the event occurred during the study observation phase except if the onset date is partially completed or other data (e.g. a stop date) indicates differently.

A subgroup analysis of tolvaptan use will be performed, investigating longitudinal changes within subgroups and differences between subgroups. In this analysis patients will be split into ‘patients with tolvaptan treatment ongoing at baseline’ and ‘patients with tolvaptan treatment not ongoing at baseline’. A subgroup of ‘patients starting tolvaptan during follow-up’ may also be included.

### Data management

Data will be collected on electronic case report forms (e-CRF). Only persons authorized by the Investigator to make original e-CRF entries are allowed to make corrections. Otsuka staff or CRO working on behalf of Otsuka will review the data entered into the e-CRFs by investigational staff for completeness and accuracy and will instruct the site personnel to make any required corrections or additions.

## Discussion

The ACQUIRE study aims to collect real-world data over the course of 18 months to develop prognostic markers and treatment response prediction models for rapidly progressing, early-stage ADPKD. By collecting data regularly over 18 months, this study aims to investigate any change in HRQoL, PROs and treatment preferences as individual patient’s ADPKD progresses. The study may use SEM to investigate possible relationships between variables of interest that could predict PRO scores as disease progresses.

### The ACQUIRE study addresses unmet needs

There is a need for data based on PROs in ADPKD, particularly during the early stages of CKD, measured by disease-specific instruments [32]. There is conflicting evidence on the impact of ADPKD on HRQoL. Some evidence suggests ADPKD patients with rapidly progressing disease have reduced HRQoL, at all disease stages, compared with the general population [2], whereas other evidence suggests that, during the early stages of disease, there is no significant difference between HRQoL in ADPKD patients and the general population [33]. These differences may be due to collecting data via general HRQoL questionnaires that do not accurately identify the impact of ADPKD symptoms. For example, previous studies have shown that HRQoL correlates most accurately with physical symptoms (such as liver and kidney volume and abdominal distention), rather than CKD stage [2, 34, 35]. Other studies have attempted to measure the impact on HRQoL using kidney disease-specific measures or unvalidated ADPKD-specific questionnaires [36–38].

This is the first study collecting data on HRQoL using validated ADPKD-specific questionnaires. These questionnaires will be the first to measure the impact of rapidly progressing early-stage ADPKD on patients’ quality of life, the impact of ADPKD progression (by comparing baseline results to endpoint disease stage) and the impact of ADPKD treatments. By using multiple questionnaires investigating different symptoms of ADPKD, this study will provide novel insights into the impact of all symptoms during rapidly progressing early-stage ADPKD. Additionally, the TSQM-9 provides information about patient’s satisfaction with current treatment; by comparing this information with HRQoL and disease burden questionnaires, relationships between treatment satisfaction, HRQoL, prognostic markers and treatment response can be investigated. The results of these analyses have the potential to improve treatment response predictors in rapidly progressing early-stage ADPKD.

This is also the first study aiming to measure ADPKD patient treatment preference using a DCE. The DCE in this study has been validated by an expert steering committee and aims to investigate the roles of treatment attributes in patient treatment choices. Mixed effect and multinomial logit models are commonly used to analyze patient DCE responses [39], as these models allow each treatment attribute to be valued independently of others and then listed in order of most to least important when patients are choosing between hypothetical treatment options, as well as allowing assessment of how patients trade-off in their decision making and the existence of any preference heterogeneity. DCEs can explore patient preferences for attributes of healthcare interventions, for example treatment efficacy and side-effects, and act as a predictive treatment tool to aid treatment decisions based on patient response. Previously, this methodology has been used to guide treatment decisions in a range of therapeutic areas, including CKD [40–42]. These studies demonstrated that CKD patients value QoL and treatments that improve the day-to-day experience of living with the disease. For example, one study found patients were willing to forgo 23 months of life expectancy if they could receive home dialysis with fewer travel restrictions [41]. Another study found ESRD patients were willing to accept a 6% increase in medication-related heart attack risk to avoid two blood transfusions per month [42].

The DCE used in this study will provide information about patient treatment preference, based on possible treatment side effects and outcomes. Specifically, the DCE was designed to compare patient tolerance of aquaresis against long-term treatment outcomes. Aquaresis is a known and expected side effect of tolvaptan treatment, and physicians highlighted this as a concern during the development of the DCE in this study. However, there is little evidence on the impact of tolvaptan-related aquaresis on patient HRQoL [10]. The results of this DCE study will provide information about the importance of aquaresis to patients compared with other long-term benefits of tolvaptan treatment, such as delay to ESRD. Additionally, preference heterogeneity in terms of patient characteristics will provide information to help predict specific patient perception of the benefits of treatments. It may thus be easier for physicians, when discussing treatment options with each patient, to predict whether a patient will value the benefits of a treatment, such as tolvaptan, while coping well with potential side effects. This may allow physicians to highlight those patients that have a diagnosis qualifying them for tolvaptan treatment, but also whose HRQoL will be most improved by tolvaptan treatment.

### Study limitations

While this study aims to provide valuable insights into the burden of early-stage ADPKD, there are several limitations that should be considered. While study results will potentially provide an indication about the real-world impact of early-stage ADPKD on patient HRQoL and patient treatment satisfaction and preference, all analyses using PRO data and the DCE are exploratory. As a result, no formal hypotheses are provided for these analyses and no firm conclusions from the analyses can be drawn. PROs will be collected remotely, with participants completing the questionnaires via a web-based data capture system. Although this was considered to be the best method of maximizing the response rate, it is still likely that a significant number of patients will not complete the questionnaires. Additionally, this may lead to selection bias as individuals with low QoL may be less able to complete the questionnaires resulting in artificially inflated scores [43]. It may also not be possible to extend the findings of this study to patients with slower progressing ADPKD. Participants have been selected because there is evidence that they suffer from rapidly progressing ADPKD and it is possible that patients with faster progressing disease may be more willing to accept treatment side effects for a relatively short delay to ESRD given that they are more likely to progress to ESRD. Finally, the attributes of the treatment alternatives presented in the DCE are hypothetical. Therefore, the results of the DCE study will be subject to potential hypothetical bias.

## Conclusions

This study is the first using validated disease-specific PRO questionnaires and a DCE to investigate the everyday burden of early-stage, rapidly progressing ADPKD on patient HRQoL. The study aims to produce reliable and relevant information about the real-world impact of early-stage ADPKD symptoms to help guide research and treatment development. In addition, the DCE may provide information about patient treatment preference that could be used, alongside discussions with individual patients, to aid physician decisions regarding patient treatment goals.

## Data Availability

Data sharing is not applicable to this article as no new data were created or analysed in this study.

## Abbreviations

ADPKD: Autosomal dominant polycystic kidney disease
ADPKD-IS: ADPKD Impact Scale
ADPKD-PDS: ADPKD Pain and Discomfort Scale
ADPKD-UIS: ADPKD Urinary Impact Scale
cAMP: Cyclic adenosine monophosphate
CKD: Chronic kidney disease
DCE: Discrete choice experiment
ESRD: End-stage renal disease
GFR: Glomerular filtration rate
HRQoL: Health-related quality of life
MCS: Mental component score
PCS: Physical component score
PKD1: Polycystin 1
PKD2: Polycystin 2
PRO: Patient reported outcome
QoL: Quality of life
SD: Standard deviation
SEM: Structural equational modelling
SF-12: 12-item Short Form Health Survey
SF-36: Short Form-36
TSQM: Treatment Satisfaction Questionnaire for Medication
UK: United Kingdom
